# Coach perspectives on coach-athlete relationships and characteristics of Generation Z academy level rugby union players from South Africa

**DOI:** 10.3389/fspor.2024.1461951

**Published:** 2024-10-21

**Authors:** Marc Landman, Heinrich Grobbelaar, Wilbur Kraak

**Affiliations:** ^1^ Division of Sport Science, Department of Exercise, Sport and Lifestyle Medicine, Stellenbosch University, Stellenbosch, South Africa; ^2^ Department of Sport, Recreation, and Exercise Science, University of the Western Cape, Cape Town, South Africa; ^3^ Department of Exercise and Sport Science, University of the Free State, Bloemfontein, South Africa

**Keywords:** coaching effectiveness, closeness, complementary, communication, coach education

## Abstract

**Introduction:**

The coach-athlete relationship is not merely a by-product of the coaching process but serves as its foundation. Coaches today must align their approaches with the characteristics (character, strengths, and growth areas) of a new generation of athletes, commonly referred to as Generation Z (Gen Z). Born between 1995 and 2012, Gen Z's grew up in a digital age, which shaped their character and behaviour. The purpose of the study was to explore coach-athlete relationships, the characteristics of Gen Z athletes that influence this relationship, and the process of building healthy coach-athlete relationships, from the perspectives of coaches.

**Methods:**

The study used a qualitative methodology to elicit the perspectives of 11 male rugby union coaches (M ± SD age: 42.09 ± 7.56 years; with 12.83 ± 3.48 years of coaching experience), through semi-structured individual online interviews. We developed the interview script from Jowett's Coach-Athlete Relationship Questionnaire (CART-Q). The interviews were analysed using Merriam and Tisdell's six-step process.

**Findings:**

The analysis yielded six super themes (and 17 themes). 1. Coach perspectives on coaching Gen Z players (developmental facilitators and debilitators, Covid-19's effect on development). 2. Closeness between coaches and athletes [loving and caring, getting closer, trusting, (not) respecting some players]. 3. Commitment (personally and towards the team, sacrifices). 4. Complementarity (goal setting, collectively working towards common goals). 5. Co-orientation (communicating, reciprocal relationship views). 6. Developing the coach-athlete relationship (approaches thereto, practical steps).

**Discussion:**

Various facets of the coach-athlete relationship are interlinked and shaped by the characteristics and behaviours of this generation of athletes. Practical recommendations are offered on how to cultivate the coach-athlete relationship on emotional, cognitive, and behavioural levels.

## Introduction

The coach-athlete relationship serves as the bedrock of success in sports, fostering an environment built on trust, communication, and mutual respect (1). Coaches play an important role in motivating and inspiring players, helping them to overcome challenges and excel individually and as a team. Effective coaching behaviours are pivotal in guiding athletes towards positive outcomes, encompassing factors such as performance, enjoyment, self-esteem, and perceived ability (2). To improve this relationship, coaches must possess a deep understanding of their athletes’ characteristics, strengths, and weaknesses (3). Likewise, contextual information about their athletes’ socioeconomic and educational background, personal needs and ambitions requires careful consideration.

Generation Z (Gen Z) refers to individuals born between 1995 and 2021. Individuals from this era have been influenced by digital technology that have shaped their character and behaviour (4). Growing up surrounded by technology, they have developed unique characteristics and preferences influenced by constant connectivity and easy access to information (4). Additionally, as the first generation born in post-Apartheid South Africa, this generation carry the complexities of changing the country's tainted socio-political landscape (5). This dual identity, influenced by both technological advancements and the historical context, contributes to the diverse perspectives and experiences of Gen Z individuals (4). Shaped by the digital age, these individuals possess distinct characteristics that necessitate coaches to adapt their approaches accordingly (6–8). For example, Gen Z's are prone to technological distraction and rapid task-switching (6), and tend to have well-developed technological skills, however, they struggle with attentional and emotional control, communication, and dealing with setbacks (8). The emergence of Gen Z athletes presents a distinctive challenge, and coaches who seek to establish meaningful connections with these individual must understand their unique characteristics (8–10). Research by Gould et al. (2020) (8) sheds light on some of these characteristics and the challenges faced by Gen Z athletes, however, uncertainty remains about how these characteristics influence the coach-athlete relationship.

Jowett's (11) Coaching Efficacy Model, commonly referred to as the 3 + 1C's Model allows an exploration of this relationship. The model comprise Closeness (emotional and personal connection), Commitment (unwavering dedication to sustain the relationship), Complimentarity (cooperation and interdependency) and Co-orientation (shared perceptions and understanding of the relationship) (12, 13).

This study is important for sports academies who identify and support promising athletes and play a pivotal role in nurturing talent and offering specialised training and opportunities. By studying the coach-athlete relationship with Gen Z rugby players, we aim to provide insights into effective coaching strategies. This research bridges gaps in understanding coach-athlete dynamics, contributing to coaching evolution and athletes’ success in a dynamic sports landscape.

This purpose of this study was to provide a comprehensive understanding of the coach-athlete relationship from the perspective of coaches working with Gen Z academy-level rugby players. The research questions were: (1) How do coaches perceive the coach-athlete relationships and characteristics of Gen Z athletes, (2) Which factors influence these perceptions? And (3) What strategies do coaches employ to cultivate healthy relationships with their Gen Z players?

## Methodology

### Research design

The study used a qualitative research design as described by Merriam and Tisdell (14), that allowed for an exploration of the subject matter, including the attitudes, beliefs, and reactions of coaches towards society and the specific topic under investigation. The COREQ checklist was used to ensure the comprehensive reporting of qualitative information (15). The study received approval from the Stellenbosch University Research Ethics Committee for Social, Behavioural, and Educational Research (REC: SBE - 26407).

### Research paradigm

The study used an interpretive epistemological approach, acknowledging that knowledge is socially constructed. The research team aimed to understand and interpret the social contexts of the coaches, capturing their nuanced perspectives and subjective experiences on the coach-athlete relationship. The study explored how participants constructed meaning in their natural settings, emphasizing what they considered relevant and important. This allowed participants to convey their experiences while the research team interpreted them. The study's ontology aligned with the idea that reality is socially constructed, varied, and unique to participants. By delving into the coaches’ perceptions of the coach-athlete relationship, the primary investigator (PI), ML, conducted the research effectively. ML, a male Gen Z researcher with a Master of Science degree in Sport Science, attended a 5-day qualitative research course at Stellenbosch University before conceptualizing the project. ML is a qualified Level 2 International Hockey Federation coach, coaching at both school and club levels, and had no prior connection with the study participants. Considering the backgrounds and social contexts of both athletes and coaches, the study aimed to yield a comprehensive understanding of the coaches’ perceptions. It recognised that these perceptions are subjective and influenced by their social context, making it essential to explore these factors. The study, through an interpretive social science lens, uncovered the rich tapestry of the coach-athlete relationship, with the PI also reflecting on his own position.

### Participants

Purposeful sampling was used to recruit 12 coaches from rugby academies in South Africa, aiming to elicit information-rich data (16). Information from 11 coaches, who coached men's rugby academies, was included in the final analysis. The information of one coach, who coached within a women's rugby academy was excluded from the analysis, to ensure a homogenous sample. To ensure confidentiality, the coaches’ names and personal details were removed from the transcriptions, and pseudonyms were used instead. Table 1 outlines the coaches’ demographic information and coaching experience. Coaches were required to have a minimum of one years of coaching experience at the academy level to ensure familiarity with working with Gen Z athletes. Ten coaches held World Rugby Level Two certificates, and one coach held a World Rugby Level Three certificate.

**Table 1:** Demographic information of the 11 participating coaches.

Demographic information	Mean ± SD age	Min-Max age
Age (years)	42.09 ± 7.56	33–59
Coaching experience (years)	12.83 ± 3.48	6–24
Coaching at academy level (years)	3.77 ± 4.68	1–11

### Data collection procedures

#### Researcher positioning

ML's chosen position for the study was aligned with Savin-Baden and Major's (17) approach, that involved three key areas: the topic, research participants, and context/process. ML is a Gen Z individual himself, whereas his co-authors are respectively Generation Y (commonly referred to as millennials) and Generation X. As the primary instrument^1^, he continuously reflected on his own worldview, and endeavoured to achieve congruency between the epistemology and ontology adopted during the study. He used a methodological journal to remain aware of his own biases and perspectives, throughout the study (see text footnote 1). Reflexivity guided his self-awareness, assessing how his views may have influenced the design, execution, and interpretation of the research findings (15). Despite personal experiences and biases informing the research, a high level of objectivity was maintained to ensure research integrity.

#### Interview questions

The data collection process involved conducting one-on-one interviews. These semi-structured interviews used a set of questions derived from Jowett's (18) Coach-Athlete Relationship Questionnaire (CART-Q), thereby providing a theoretical framework for analysing the coach-athlete relationship dynamics.

#### Interviews

Before the interviews, ML conducted three pilot interviews and consulted with HG and WK for feedback. The participants received pre-interview packages, in which “Gen Z” was replaced by “academy players” or “current academy players” for consistency. The rationale for this deception is explained later. Informed consent was obtained from all participants. Online interviews were conducted via MS Teams, whilst confidentiality were ensured through data de-identification. The 11 semi-structured interviews, that lasted one hour and twenty-three minutes on average (range: 56–117 min), explored coach-athlete relationships with Gen Z athletes. There were no follow-up interviews during data collection. The term “current academy players” was used instead of “Gen Z” to prevent stereotype influence, following the recommendations of the ethics committee. Whenever a participant referred to the term “Gen Z”, they were informed that this was indeed the population that this study set out to explore, and the reason for this deception was explained (i.e., preventing stereotypical responses). Participants who did not use this term was informed about the deception and the reasons for it on conclusion of the interview.

### Data analysis

The recorded interviews were transcribed verbatim. Rigor and enhanced trustworthiness were achieved through two distinct processes. ML as the primary data collection instrument, performed manual analysis as suggested by Czech (see text footnote 1) Guided by Merriam and Tisdell's (14) six-step process, the elicited information was analysed by (1) identifying relevant segments of transcriptions, (2) coding the information, (3) grouping the codes into categories, (4) naming these categories, (5) narrowing the focus of the categories, and (6) refining the analysis to ensure that the research questions were adequately addressed. ML maintained a journal to establish an audit trail, with field notes scribbled down during interviews and documenting decisions throughout the analysis and interpretation. This included regular check-ins and comparisons with his supervisory team, who questioned the procedures, analysis and interpretations. Throughout the data collection and analysis, ML bracketed his own beliefs and perceptions, although a sense of subjectivity remained, because of the difficulty to completely bracket out one's biases. The findings of the study were shared with the participants after the completion of data analysis and interpretation as a form of member checking.

## Findings and discussion

The data analysis generated six super themes (Characteristics of Gen Z, Closeness, Commitment, Complementarity, Co-orientation, and Developing the coach-athlete relationship) and 17 themes, as depicted in Table 2.

**Table 2:** The super themes, themes and selected sample quotes.

Super themes	Themes	Sample quotes
1. Coach perspectives on coaching Gen Z players	Developmental facilitators	“The players have become better and more effective at problem solving.”
	Developmental debilitators	“Gen Z athletes want to be the heroes in their own stories, they tend to make excuses and shoulder the blame.”
	Effects of COVID-19 on player development	(Gen Z's) “feel very awkward without their phones, they may have traded physical interactions for technology.”
2. Closeness	Loving and caring	“Caring is the key to developing players.”
	Getting closer	“…is being able to share openly; it is developed over time, and spending time together, and having experiences together.”
	Trusting	“It is about putting in effort both ways, it is about trusting and believing in the person.”
	Respecting some players	“Some athletes have decided they do want to get better, so you appreciate and respect their efforts, some haven’t so you don’t respect their efforts and energy.”
	Not respecting some players	
3. Commitment	Committing personally	“If you demand commitment from your players, you must be committed first.”
	Committing towards the team	“We are committed because we have the same goals in mind, if we have the guys all working in different directions and for themselves, it is a recipe for disaster.”
	Sacrifices	“Those guys who lay out everything and grind to get better, I appreciate their sacrifices.”
4. Complementarity	Goal setting	“Individual goals are very important and aligning those individual goals with your team goals, which is important.”
	Collectively working towards a common goal	“There needs to be alignment with everyone for effective goal setting and maintaining good relationships.”
5. Co-orientation	Communicating	“As a coach, I need to narrow down to three things, the players can’t process more than that.”
	Reciprocal relationship views	“The players need to be seen as individuals before anything else.”
6. Developing the coach-athlete relationship	Approaching the coach-athlete relationship	“It is about the person first and foremost, before the athlete or player.”
	Practical steps	“One-on-one discussions and time together to build relationships are vital.” “Arriving early and staying late”

**Table 3:** Super theme 1: Characteristics of Gen Z academy-level rugby players from the coach's perspectives.

Themes	Sub-themes	Categories of codes	Codes
Developmental facilitators	High emotional intelligence	Good at solving problems	
		Knowing how to take care of themselves	
		Knowing their opinions count	
	Goal orientated	Results driven	
		Wanting to be part of the learning process	
	Technological savvy	Clued up on recent research	
		Understanding the process of development	
	Having a good support structure		
Developmental debilitators	Taking feedback very personally		
	Feeling entitled	Making excuses	Not my fault we lost
		Medals for participating	Everyone must be a winner
		Not being honest with themselves	
		Needing to be the Heroes in their stories	
	Mentally and physically soft		
	Instant gratification	Quitting easily	
		Expecting results immediately	
		Struggling with commitments	
		Not understanding the slow process of improvement	
Effects of Covid and missing two years of rugby	Physically		
	Emotionally		

## Super theme 1: Characteristics of Gen Z

Table 3 presents the developmental facilitators and debilitators as well as the effects of Covid and missing two years of rugby. In today's dynamic sport coaching landscape, adapting ones coaching approaches to best suit the unique attributes of Gen Z athletes has become important (19). Technological advancements and global events has moulded this generation's social landscape (20).

### Developmental facilitators

Coaches noted high emotional intelligence among Gen Z athletes, who prioritise their individuality and understand the importance of managing emotions for long-term success. They excel in self-awareness and problem-solving, actively engaging in discussions about challenges, as supported by earlier research (4, 8, 21). Additionally, these athletes are increasingly outspoken, valuing their own experiences and knowledge, as indicated by Kompa.^2^ They place a premium on their learned experiences and their own knowledge.

Coach Mike mentioned that: “*The old-school taskmaster approach doesn*’*t work so well with Gen Z athletes as they don*’*t respond, they struggle with the top-down leadership model as it doesn*’*t make affordances for the players and for their knowledge*.”

Coaches who encourage this openness foster stronger player engagement, echoing the findings of Gould et al. (8) and Kraak (4) on the importance of empowering athletes in their developmental plans. Gen Z athletes exhibit a strong orientation towards goals, driven by self-confidence and a desire for tangible results, aligning with Gould et al.'s (8) findings on their goal-directed nature. Moreover, coaches recognise their tech-savvy tendencies, integrating digital tools like video analysis into training, consistent with Gould et al.'s (8) observations. Coach Chris added: “*these guys are incredibly good with technology, as coaches we must harness that ability*.” This generation's adeptness with technology allows them to access and utilize information more comprehensively, enhancing their developmental process and pursuit of goals. Gen Z athletes prefer a democratic style where they are part of the process and prefer quick feedback using video analysis following a performance (4).

### Developmental debilitators

Coaches noted various challenges faced by Gen Z athletes, including negative feedback internalisation and attention span struggles, echoing Rothman's (9) observations. Entitlement also emerged as a hurdle, with athletes seeking recognition regardless of their performance, in line with Gould et al.'s (8) findings. Coach Josh highlighted this, noting the players’ desire to be the: “*heroes in their own stories*,” often at the expense of acknowledging their shortcomings. The fear of failure and the need for immediate gratification complicate coaching, as discussed by Turner (22). Coach Scott emphasised the athletes’ “*reluctance to admit their training efforts*,” affecting their selection for starting lineups. This reluctance, influenced by the desire for success and professional contracts, underscores the importance of effective communication in selection processes, as emphasized by Rothwell (23). Open channels of communication are crucial for addressing athletes’ concerns and fostering their development effectively.

### Effects of COVID-19 and missing out on two years of development

Coaches working with Gen Z athletes must address the challenge of a two-year developmental setback due to the Covid-19 pandemic and subsequent lockdowns. Physically, players missed crucial opportunities for skill development, face-to-face coaching, and competitive play, requiring coaches to elevate their players back to pre-pandemic standards (24). Emotionally, athletes crave meaningful connections after extended isolation, aligning with Ricks’^3^ observations. This need may lead to a reliance on technology, as noted by coaches observing athletes feeling: “*uncomfortable without their phones*,” reflecting Hawkins’ (25) findings on Gen Z's high screen time usage. Recognising this reliance, coaches must adapt, leverage technology to foster meaningful connections and enhance coaching effectiveness amidst the evolving landscape shaped by the pandemic's impact.

## Super theme 2: Closeness

Table 4 presents the factors identified by coaches, categorised into themes such as loving and caring, getting closer, trusting, and respecting.

**Table 4:** Super theme 2: Closeness, the 1st C from the coach's perspectives regarding the coach-athlete relationship with Gen Z academy-level rugby players.

Themes	Sub-themes	Categories of codes	Codes
Loving and caring	Perception of feeling loved	Need to know they are loved and cared for	
	Effective communication	Important to foster closeness	
		Sharing technical and psychological aspects with players	
Getting closer	Continuous journey	Everyday interactions	Not a set moment or big occasion
	Honesty and openness	Gen Z's need authenticity	It enhances the relationship if all parties are authentic
		Consistency	Provides security
			Promotes trust
Trusting	Mutual effort	Two-way street	Daily commitment
	Communication	Creating a safe space	Opportunity to share stories and experiences
Respecting	Respecting some players	Those who are committed and dedicated	Not based on financial compensation
	Not respecting some players	Those who do not put in effort and time	Lack of care/effort

### Loving and caring

The athlete's perception of feeling valued and loved by the coach significantly influences the development of the coach-athlete relationship, as outlined by Jowett and Poczwardowski (26). LaVoi's (27) research emphasises the importance of communication in fostering closeness within this relationship. Effective communication not only conveys technical information but also addresses psychological aspects; motivating and reassuring athletes. Coaches, as noted by Jowett and Nezlek (13), fulfil various roles including mentors, motivators, and confidants, actively engaging athletes in their own development. Coach Tim added: “*young players need your attention, they need your time.”* To foster closeness in your coach-athlete relationship, time and attention are vital commodities and are important for both parties to keep valuing the relationship and getting closer to each other.

### Getting closer

Gu et al. (28) suggested that coaches who develop close relationships with athletes offer tailored guidance based on their individual needs, personality, and athletic abilities, aiding their advancement in training and competition. In line with the principles of positive psychology, organisational psychologists suggest that engagement is the conceptual opposite of burnout, characterised by a state filled with positive emotions and motivations (29). Lonsdale introduced this concept into the field of sport, highlighting that athlete engagement reflects a lasting and positive cognitive and emotional experience in sport (30). Building a close coach-athlete relationship is a continuous journey throughout the season.

This closeness is cultivated through everyday interactions, such as gym sessions and meetings, and extends beyond a professional connection to a personal bond forged through shared experiences. Coach Ryan added: “*closeness is being able to share openly; it is developed over time, and spending time together, and having experiences together*.” Honest and genuine conversations, as recommended by Coach Steve, are crucial for Gen Z athletes who value authenticity. Jowett and Poczwardowski (26) highlighted honesty and communication as essential attributes for enhancing this relationship. Consistency is also key, ensuring coaches are dependable and present, providing a sense of security for players, as noted by Coach Scott: “*the players need to know what to expect from you.”* This consistency fosters trust and understanding, particularly important at the beginning of the season.

### Trusting

Mayer et al. (31) defined trust as “*the willingness of a party to be vulnerable to the actions of another party based on the expectation that the other will perform a particular action important to the trustor, irrespective of the ability to monitor or control that other party*” (p. 712). Thus, trust is the positive expectation and willingness of the trustor to be vulnerable. In the context of direct and repeated interactions between coaches and athletes, if athletes believe that the coach will fulfil their commitments to the relationship, they will trust the coach more (32).

Trust in the coach affects the performance of collegiate basketball teams (32) and moderates the relationship between gratitude and self-esteem (33). “*It is about putting in effort both ways, it is about trusting and believing in the person*” says Coach Mike. The quote emphasises the mutual effort required for building trust in the coach-athlete relationship, particularly emphasising trust in the person before the athlete. This trust is nurtured through consistent effort and shared experiences, as discussed by Seemiller and Grace (7). Effective communication plays a crucial role in fostering trust, echoing findings by Shatto and Erwin (10) on the importance of communication for Gen Z athletes. The coaches stressed the need for athletes to feel comfortable sharing personal stories, highlighting the daily commitment required to develop trust. Coach John confirms this thought and shares that: “*the players need to be able to share openly with you as a coach and confide in you*.” Additionally, coaches recognise the importance of consistency and organisation in demonstrating reliability and commitment to the athletes’ development, further solidifying trust in the coaching relationship.

### Respecting

The coaches’ responses regarding respect revealed two sub-themes: respecting some players while not respecting others. Certain coaches noted that respect depended on players’ dedication to development, with their commitment wavering when they are not financially compensated. Coach Ryan noted: “*the commitment and efforts may waiver as these players don't get paid*.” Conversely, investing in players’ lives and demonstrating care fosters mutual respect, aligning with Gen Z athletes’ desire for authentic connections and the importance of loving and caring in relationship-building. Song et al. (34) suggests that coaches who develop close relationships with athletes offer tailored guidance based on their individual needs, personality, and athletic abilities, aiding their advancement in training and competition. When athletes experience both vitality and learning, they enter a thriving state, characterized by a desire for development, progress, and breakthroughs. This drive fosters athletic engagement in both training and competition. Cultural differences also influence respect dynamics, emphasising the need for coaches to understand and respect the athletes’ backgrounds. Coach Derek added: “*every single culture is different, and every cultural journey is different, we can't employ a one-size fits all approach.”* However, some coaches prioritise winning over holistic player development, leading to a lack of respect for their athletes’ well-being. This disregard for holistic player development may negatively impact the coach-athlete relationship, as coaches’ behaviours influence their athletes’ motivation (35). Coaches should lead by example, fostering autonomy and competence in players to earn their respect through mutual effort and understanding.

## Super theme 3: Commitment

Table 5 highlights the mutual dedication and unwavering commitment between coaches and athletes. This joint investment of time and effort amplifies motivation and satisfaction within the relationship (12, 13). Coaches emphasised the importance of personal commitment and dedication to team success, as well as the sacrifices involved. Coach Steve stressed the role of commitment in driving success, reflecting the significance of this aspect in coaching. *“As a coach, you can never say to your players, listen I cannot make this or make it here, unless it is an absolute crisis. If you demand commitment from your players, you must be committed first. If you are committed wholeheartedly and completely in the process, you will draft commitment from them too.”* Coach Scott shared: “*if I don't make the sacrifices and don't put out clear goals for the players, then I can't expect them to achieve their goals, yes, again for me it's not a 50/50 thing it's a 100 thing, if I give my 100 then I expect their 100 in return.”*

**Table 5:** Super theme 3: Commitment, the 2nd C from the coaches’ perspectives with Gen Z academy-level rugby players.

Themes	Sub-themes	Categories of codes	Codes
Committing personally	Alignment of team and personal goals	So players do not feel they are working against their coaches	
	Reciprocal commitment	Coaches committing first	
		Players slowly joining in	
	Importance of trust	Players will commit if they can trust the coach	
Committing to the team objectives	Collective goals	Alignment of team and personal goals	Discipline to achieve goals
	Fostering a motivational climate	Focusing on development and learning	
		Avoiding use of fear and intimidation	
Sacrifices	Personal sacrifices	Sacrificing social opportunities	
	Coaches sacrifices	Coaches sacrificing first	
	Appreciating players efforts	Their time and efforts	

### Committing personally

Commitment signifies the willingness of coaches and athletes to forge and uphold cooperative relationships, highlighting their psychological inclination to unite for shared interests (28). Athletes with strong commitment demonstrate the courage to express their needs and values, facilitating the formation of an equitable, voluntary, inclusive, and progressive community of interests (36). The theme of committing personally emphasises the importance of alignment between coaches and athletes in pursuing team goals. Coaches play a pivotal role in setting the commitment standard, demonstrating initiative, and showing commitment to understanding athletes. Coach Scott noted that commitment from coaches fosters reciprocal commitment from athletes. This commitment, as Coach Mike highlighted: “*involves loving and caring for players even when recognition is not guaranteed”* and “*fostering a sense of mutual investment.”* Kao et al. (37) underline the significance of trust in fostering commitment, as athletes are more likely to invest in the relationship if they trust their coach. This reciprocal commitment creates a positive cycle where coaches are motivated to invest more in athletes as they see the impact of their guidance, enhancing athlete performance and strengthening the coach-athlete bond.

### Committing to the team objectives

Drawing from strong culture theory, organisational commitment stems from the collective goals and internal acknowledgment within the team. When team members share cohesive objectives and receive organizational recognition, it fosters heightened job satisfaction and work engagement (38). A prevalent theme in the study is the commitment towards team objectives, with both coaches and athletes dedicating themselves to collective goals. Coach Josh added: “*we are committed because we have the same goals in mind, if we have the guys all working in different directions and for themselves, it is a recipe for disaster.”* Discipline was highlighted as essential for maintaining commitment and creating a supportive environment for Gen Z players to succeed collectively and individually. Despite challenges such as a lack of financial incentives, coaches emphasize the importance of being present, connected, and invested in players to maintain strong commitment. Coaches can enhance player commitment by fostering a positive motivational climate focused on learning and development rather than fear or intimidation (39). Effective communication, transparency, and trust are crucial for maintaining commitment (see text footnote 2) (40). Additionally, coaches appreciate the sacrifices made by players who demonstrate exceptional effort and dedication, while expressing dissatisfaction with those who fail to prioritize team commitments.

### Sacrifices

Research indicates that effective leadership positively influences team members’ efforts (41). For example, when team leaders employ effective leadership methods and team members willingly make sacrifices for the collective, teams are more likely to achieve their goals (41). Sacrifice, as described by Prapavessis and Carron (42), involves group members voluntarily giving up privileges for others’ benefit, encompassing concepts like empathy, altruism, cooperation, and loyalty. Within the context of sports, sacrifice during daily practice and competition are termed “inside sacrifice” (43), comprising both personal and teammates’ sacrifices. As teams operate within a collective context, understanding personal sacrifice and expected behaviours among team members is crucial (43). Decades of research have linked sacrifice with group processes, particularly within sports, where it correlates strongly with coach leadership, coach-athlete relationships, and athlete leadership (43). Inside sacrifice reflects both effort and the impact of effective athlete leadership. When players perceive supportive leadership that coordinates actions and enhances performance, they are more inclined to exert effort for the team. Consequently, leaders who effectively persuade and support teammates may elevate inside sacrifice within the team (41). The theme of sacrifices in athlete development revealed three sub-themes, starting with personal sacrifice.

Becoming a high-level rugby player demands substantial commitment, including sacrifices in social life, studies, and employment opportunities. This dedication extends beyond the field, encompassing mindset, nutrition, training, and performance review. However, not all academy level players will reach the elite level. Still, they need to sacrifice if they want a chance of success, despite the inherent risks thereto. Coaches emphasise that their sacrifices aim to support players’ growth and development, illustrating their commitment to player advancement. Coach Scott added: “*If I sacrifice everything because we must make sacrifices. So, I sacrifice everything to help them achieve their goals, then I expect them to do everything in their power to achieve that goal.”* Additionally, coaches appreciate players who demonstrate exceptional effort and commitment, including injured players who attend training sessions to contribute. Coach Ryan highlighted his appreciation for: “*injured players who attend training, even if they cannot actively participate, but are just there to help in the session.”* However, coaches’ express disappointment in players who fail to prioritize their development, citing instances of weak excuses for missing training sessions, echoing sentiments from the coaches’ experiences at higher levels of play.

## Super theme 4: Complementarity

Table 6 emphasizes shared objectives, effective communication, and a positive atmosphere, fostering optimal performance and holistic development as part of Complementarity (11, 12).

**Table 6:** Super theme 4: Complementarity, the 3rd C from the coaches’ perspectives working with Gen Z academy-level rugby players.

Themes	Sub-themes	Categories of codes
Goal setting	Gen Z Players	Focus more on outcomes than processes Want instant gratification Fear failure Impacted by parents
	Coaches	Develop a growth mindset Create a culture of learning in the team
Collectively working towards a common goal	Shared goals	Based on a good relationship between coaches and players. Explain the rationale behind drills or decisions. Employ a democratic approach

### Goal setting

The table presents factors identified by coaches, including goal setting and collaborative efforts toward shared goals, as identified by Jowett (44). Gen Z athletes prioritise goal achievement, emphasizing the alignment of personal and team goals to foster cooperation, as noted by Coach Derek who highlights the importance of this alignment. Coaches stress accountability and support in helping athletes achieve their goals, reflecting Gould et al.'s (8) findings on Gen Z athletes’ focus on outcome goals. Transparency and collaboration in goal setting are emphasized to minimise disagreements, supported by Felton et al. (45) and Martindale et al. (46). However, coaches face challenges in goal setting with Gen Z athletes, who often struggle with realistic expectations and prioritise outcomes over the developmental process. This tendency, influenced by cultural and parental factors, impedes long-term commitment to goals (47). To address these challenges, coaches must shift athletes’ focus to a growth mindset, emphasising learning, and resilience over immediate success (48). Creating a culture of learning, where feedback is valued and mistakes are seen as opportunities for growth, is essential in fostering development-oriented approaches in Gen Z athletes.

### Collectively working towards a common goal

Successful collaborations between athletes and coaches hinges on working towards shared objectives (11). Coaches stress the importance of fostering relationships wherein Gen Z athletes feel valued and capable of contributing meaningfully to the team, whether on the field or during discussions. To achieve this, coaches emphasise the need to explain the rationale behind drills or tactics, a strategy echoed by Gould et al. (8) to enhance athlete motivation. Coach Scott agreed and shared: *“If you go and explain the why to the players, listen it is important that you do this on this side of the field, otherwise we will not go on that side of the field, and you show them examples. Then he is in it to achieve the goal! However, if they do not know the why, then they are not fully committed to achieving those goals.”* Supporting autonomy and explaining the reasoning behind decisions aligns with the earlier findings on autonomous-supportive coaching (49). Jowett and Poczwardowski (26) and Jowett and Kanakoglou (12) underscore the effectiveness of a democratic coaching style in nurturing successful coach-athlete relationships. Positive coach leadership (50), fosters collective efficacy among elite youth athletes, indicating the significance of coach-athlete relationships in achieving shared goals.

## Super theme 5: Co-orientation

Table 7 reveal that alignment enhances the effectiveness of the relationship between coaches and their athletes. The table presents themes on communication and reciprocal relationship perspectives from both coaches and athletes.

**Table 7:** Super theme 5: Co-orientation, the 4th C from the coaches’ perspectives working with Gen Z academy-level rugby players.

Themes	Sub-themes	Categories of codes	Codes
Communicating	On-field	Less is more	
		Take into consideration your layers background and cultures	
	Off-field	Cater for Gen Z's strengths and preferences	Animations and videos
			Use key points Avoid singling out players Check for understanding
Reciprocal relationship views	Feeling valued	Listen to ideas Open dialogue Explain your decisions as coach	
	Leveraging diverse perspectives	Enhance buy-in Integrate everyone's strengths Make players feel unique and that they are adding value	

### Communicating

Effective communication between coaches and athletes is crucial for team environment development and maintenance (51). Coaches emphasise the importance of understanding athletes’ culture for on-field communication, advocating for a concise “less is more” approach. Coach Ryan shares: “*just break it down, too much information and they won't get it, I focus on one or two things only.”* Off-field communication strategies cater to Generation Z athletes’ technology-savvy nature, utilizing animations, videos, and key takeaways to convey messages effectively. Consistent engagement with information enhances understanding, as highlighted by coaches, who stress the need to repeat key points throughout the week. However, coaches must also be mindful of how Gen Z athletes may feel singled out if questioned on unfamiliar topics, affecting their confidence. The importance of checking for understanding, especially with athletes who may not catch on quickly, underscores the mutual responsibility in the coach-athlete relationship, as perceptions from both parties contribute to its quality.

### Reciprocal relationship views

The second theme of Co-orientation delves into coaches’ and athletes’ perceptions of their relationship, crucial for successful coaching, as emphasised by the coaches. Gen Z athletes must feel valued and appreciated, with their contributions recognised and their importance to the team acknowledged. Coach Fred shared: *“You need to value and connect with the person first, then the performance and then they will move mountains for you.”* Coach Steve highlighted the importance of listening to players’ ideas and making them feel valued through open dialogue, emphasising the educational opportunity it presents. Coach Scott stressed the significance of explaining decisions and accepting mistakes to maintain player engagement and respect, he says: “*If you disagree with a player's idea and do not explain why, then you will lose the player and he will not be invested anymore. However, if you are wrong as a coach, you must accept it and you will earn their respect.”* Coaches emphasised the need for innovation and playing to Gen Z athletes’ strengths to maximise their potential, leveraging diverse perspectives for enhanced training sessions and buy-in to the coaching system. This approach aligns with Gen Z's desire to be seen as individuals, presenting a challenge for coaches to integrate these strengths effectively.

## Super theme 6: Developing the coach-athlete relationship

Table 8 presents key practical insights on the development of the coach-athlete relationship.

**Table 8:** Super theme 6: Developing the coach-athlete relationship with Gen Z academy-level rugby players from the coaches’ perspectives.

Themes	Sub-themes	Categories of codes	Codes
Approaching the coach-athlete relationship	On-going process	Relationships are built over time It takes time and dedication	
	Person 1st approach	People before players	
		See everyone as unique. Promotes player investment. Improves communication	
Practical steps	Spending time together	Meaningful time	Away from training field
		Arrive early and stay late	Create opportunity for informal interactions
		Sharing stories	Share personal stories

### Approaching the coach-athlete relationship

Coaches prioritise nurturing the coach-athlete relationship, recognising it as an ongoing process that evolves through consistent dedication and effort. Emphasising a “*person first”* approach, coaches delve into understanding each player's unique background, culture, and personal history, tailoring interactions accordingly. This approach fosters effective communication and enhances players’ investment in the coach and team principles. Furthermore, coaches underscore the importance of genuinely caring for players, acknowledging Gen Z athletes’ need for authentic care and concern. This genuine care not only strengthens the coach-athlete connection but also establishes a meaningful relationship beyond their roles.

### Practical steps

The coaches emphasised practical steps to nurture the coach-athlete relationship, such as spending meaningful time together away from the rugby fields. These interactions centred on personal conversations beyond rugby, building trust and rapport, making coaches more approachable and fostering a supportive environment. Engaging in activities like serving others and coaches Josh's approach of “*arriving early and staying late”* allows coaches to transcend their roles and become mentors, nurturing positive relationships with players off the field. Informal interactions after training sessions, like collecting equipment together or sharing experiences, are also valued for building bonds and gaining insight into players’ lives. These practices contribute to a deeper understanding and connection between coaches and athletes, enhancing the overall coach-athlete relationship.

## Practical implementation

The insights from the coaches’ perceptions and the factors influencing the coach-athlete relationship have been distilled into three key practical recommendations as presented in Table 9. Firstly, working effectively with Gen Z athletes, secondly enhancing the coach-athlete relationship, and finally key ideas for developing the coach-athlete relationship.

**Table 9:** Key findings and strategies for practical implementation.

Strategies for maximising Gen Z's potential	
Key finding	Strategy
Enhancing the facilitators of Gen Z: Emotional intelligence, goal orientation, and technology savviness	- Integrate emotional intelligence exercises during training sessions. - Implement regular reflective sessions for emotional expression and management. - Develop personalised goal setting plans aligning individual aspirations with team objectives. - Encourage short-term and long-term goal creation for motivation. - Incorporate sports technology for data-driven insights. - Establish a peer mentorship program for tech-savvy athletes.
Minimizing the effect of the debilitating factors of Gen Z: Entitlement and instant gratification	- Introduce a rewards system emphasising effort, perseverance, and improvement. - Organise workshops on the value of hard work, featuring examples of success through sustained effort. - Implement motivational sessions focusing on delayed gratification. - Encourage constructive feedback that emphasises the journey of improvement.
Strategies for enhancing the four super themes of the coach-athlete relationship	
Key finding	Strategy
Closeness	- Initiate a two-way avenue of loving and caring. - Conduct regular one-on-one check-ins for personal and athletic discussions. - Encourage athletes to express perspectives on team dynamics. - Organise off-field team-building activities. - Facilitate collaborative decision-making processes.
Commitment	- Develop individualised commitment plans aligning athlete goals with team objectives. - Recognise and commend athletes who show exceptional commitment.
Complementarity	- Integrate relationship-building exercises into goal-setting sessions. - Establish mentorship programs for experienced athletes to guide newcomers.
Co-orientation	- Implement a dual-channel communication approach for on-field tactics and off-field personal development. - Conduct communication workshops to bridge generational gaps.
Strategies for developing the coach-athlete relationship	
Key finding	Strategy
“Person-first” approach	- Encourage coaches to spend informal, non-training time with athletes to enhance personal connections. - Implement mentorship programs focusing on athletic and personal development.
Implications and recommendations	- Tailored training and coaching education for South African coaches. - Develop specific programs incorporating cultural sensitivity. - Create awareness campaigns for policymakers.
Continuous personal development	- Continuous professional development through peer coaching. - Mentorship for new coaches regarding the coach-athlete relationship. - Review and observation of other coaches’ sessions. - Coaching clinics and weekends for knowledge sharing. - Team teaching with coaches from different academies.

### Generation Z athlete development

Practical insights from coaches stress the importance of capitalising on Gen Z athletes’ strengths while addressing their challenges. Leveraging their emotional intelligence, goal orientation, and tech-savviness may enhance learning. Creating a culture of hard work and motivation may assist in managing obstacles and maximizing the athletes’ potential.

### Fostering a coach-athlete relationship

The coaches emphasized the crucial role of nurturing the coach-athlete relationship, focusing on practical strategies in line with Jowett's 3 + 1C's model. These included fostering closeness through team-building activities, promoting personal commitment among players, setting relationship-centric goals, and prioritizing effective communication with Gen Z athletes.

### Developing the coach-athlete relationship

The coaches proposed three methods to enhance the coach-athlete relationship. Firstly, they endorsed a “person-first” approach, acknowledging players as individuals beyond their team roles. Secondly, they emphasised the necessity of educating coaches about the players’ contexts and cultures, suggesting formal uptake into sports policy for optimal development. Lastly, they advocated for continuous development programs, citing guidelines by Callary et al. (52), that include diverse curriculum themes, coach portfolios, and mentorship. In settings, where formal education may not be viable for all coaches, informal methods like weekend workshops, webinars, and peer-to-peer coaching during coaching camps could facilitate knowledge exchange and relationship-building among coaches.

## Conclusions

Effectively guiding the development of Gen Z athletes requires a nuanced approach that capitalises on their unique attributes while addressing potential obstacles. Coaches can empower Gen Z athletes by focusing on their emotional intelligence, goal orientation, and technology savviness while mitigating factors such as entitlement and instant gratification that may impede long-term growth. Establishing healthy coach-athlete relationships characterised by *Closeness*, *Commitment*, *Complementarity*, and *Co-orientation* are fundamental to successful coaching. Adopting a “Person-first” approach and gaining insight into the athletes’ context and culture is crucial for nurturing this relationship. Continuous personal development ensures coaches remain adaptable and responsive to the evolving needs of Gen Z athletes. While the current study contributes to our understanding of the coach-athlete relationship, it is not without limitations. Firstly, a language barrier was encountered as the principal investigator lacked proficiency in Afrikaans, and although all the coaches were fluent in English, some preferred their native language. Secondly, the study only considered the perspective of coaches, thus providing a singular viewpoint. Thirdly, the online interviews was restrictive, especially for some of the coaches who were less familiar with modern technology, thereby reducing the richness of the information.

Recommendations for future research include revisiting aspirational disillusionment through interviews with both coaches and athletes from the same teams to understand their perceptions and experiences. Longitudinal studies could explore cyclical changes in the coach-athlete relationship, examining how it evolves. Exploring the influence of cultural backgrounds on the coach-athlete relationships, particularly in the South African context, may provide insights into the impact of cultural diversity on sporting dynamics.

## Data Availability

The raw data supporting the conclusions of this article will be made available upon reasonable request from the corresponding author.

## Footnotes

^1^Czech DR. The experience of Christian Prayer in Sport: An Existential Phenomenological Investigation. Knoxville: The University of Tennessee (2001).

^2^Kompa N. Generation Z, employee engagement and leadership communication behaviours (all theses and dissertations). 228. Portland: The University of New England (2019). p. 1–114.

^3^Ricks JR. First-generation college student success in higher education (Unpublished doctoral dissertation). North Carolina State University, Raleigh, NC, United States (2016).
